# Toward Biofabrication of Resorbable Implants Consisting of a Calcium Phosphate Cement and Fibrin—A Characterization In Vitro and In Vivo

**DOI:** 10.3390/ijms22031218

**Published:** 2021-01-26

**Authors:** Tilman Ahlfeld, Anja Lode, Richard Frank Richter, Winnie Pradel, Adrian Franke, Martina Rauner, Bernd Stadlinger, Günter Lauer, Michael Gelinsky, Paula Korn

**Affiliations:** 1 Centre for Translational Bone, Joint and Soft Tissue Research, University Hospital Carl Gustav Carus, Faculty of Medicine of Technische Universität Dresden, Fetscherstr 74, 01307 Dresden, Germany; tilman.ahlfeld@tu-dresden.de (T.A.); anja.lode@tu-dresden.de (A.L.); richard_frank.richter@tu-dresden.de (R.F.R.); michael.gelinsky@tu-dresden.de (M.G.); 2 Department of Oral and Maxillofacial Surgery, University Hospital Carl Gustav Carus, Faculty of Medicine of Technische Universität Dresden, Fetscherstr 74, 01307 Dresden, Germany; winnie.pradel@uniklinikum-dresden.de (W.P.); adrian.franke@uniklinikum-dresden.de (A.F.); guenter.lauer@uniklinikum-dresden.de (G.L.); 3 Division of Endocrinology, Diabetes and Bone Diseases, Department of Medicine III and Center for Healthy Aging, University Hospital Carl Gustav Carus, Faculty of Medicine of Technische Universität Dresden, Fetscherstr 74, 01307 Dresden, Germany; martina.rauner@uniklinikum-dresden.de; 4 Clinic of Cranio-Maxillofacial and Oral Surgery, Center of Dental Medicine, University of Zurich, Plattenstr 11, 8032 Zurich, Switzerland; bernd.stadlinger@zzm.uzh.ch; 5 Department of Oral and Maxillofacial Surgery Charité, Universitätsmedizin Berlin, Corporate Member of Freie Universität Berlin, Humboldt-Universität zu Berlin and Berlin Institute of Health, Augustenburger Platz 1, 13353 Berlin, Germany

**Keywords:** cleft alveolar osteoplasty, 3D printing, bone, bone tissue engineering, calcium phosphate cement, fibrin, mesenchymal stem cells, rat model, biofabrication

## Abstract

Cleft alveolar bone defects can be treated potentially with tissue engineered bone grafts. Herein, we developed novel biphasic bone constructs consisting of two clinically certified materials, a calcium phosphate cement (CPC) and a fibrin gel that were biofabricated using 3D plotting. The fibrin gel was loaded with mesenchymal stromal cells (MSC) derived from bone marrow. Firstly, the degradation of fibrin as well as the behavior of cells in the biphasic system were evaluated in vitro. Fibrin degraded quickly in presence of MSC. Our results showed that the plotted CPC structure acted slightly stabilizing for the fibrin gel. However, with passing time and fibrin degradation, MSC migrated to the CPC surface. Thus, the fibrin gel could be identified as cell delivery system. A pilot study in vivo was conducted in artificial craniofacial defects in Lewis rats. Ongoing bone formation could be evidenced over 12 weeks but the biphasic constructs were not completely osseous integrated. Nevertheless, our results show that the combination of 3D plotted CPC constructs and fibrin as suitable cell delivery system enables the fabrication of novel regenerative implants for the treatment of alveolar bone defects.

## 1. Introduction

Cleft lip and cleft alveolus without and with cleft palate belong to the most common congenital craniofacial anomalies in humans. Their development is caused by a malfunction during tissue fusion in embryologic development of the craniomaxillofacial anatomy. Usually alveolar clefts are treated by primary and especially by secondary bone grafting before teeth eruption [1]. However, this means that autologous bone grafting material, for example harvested from the iliac crest, is needed and that the patients (particularly young children) have to withstand two operations. Due to this reason, tissue engineering of bone grafts might be a reasonable alternative for the clinical care of alveolar cleft patients, as it avoids one surgical intervention.

Herein, we aimed to investigate the suitability of a tissue engineered implant consisting of two clinically approved materials. The first material was an α-tricalcium phosphate-based calcium phosphate cement (CPC), which transforms into nanocrystalline hydroxyapatite (HAp) [2]. Unlike common powder-liquid calcium phosphate cements, this CPC formulation consists of a mixture of precursor powders and a hydrophobic but biocompatible carrier liquid, which is exchanged with water after implantation thereby starting the setting process. This allows for limitless injectability through a nozzle and remarkably also for extrusion-based additive manufacturing, namely 3D plotting [3]. The plotting process is based on the principle of strandwise extrusion of a pasty material onto a stage. The material strands can be plotted into layers achieving a macroporous scaffold. The scaffolds being plotted from the CPC can achieve filigree shapes with a high control about pore size and pore shape [4]. Further, CPC can easily be fabricated into patient-specific shapes such as a scaphoid bone [5] or even a defect-specific implant for a cleft alveolus [6] taking usage of multi-material plotting of CPC in combination with fugitive support inks which allow for plotting of overhanging structures.

Recently we investigated the suitability of 3D plotted CPC scaffolds for the treatment of artificial alveolar clefts in a rat model [6]. Rat mesenchymal stromal cells (rMSC) were seeded onto plotted CPC structures. Our observations in vitro evidenced that rMSC were able to attach to the CPC surface, proliferate and differentiate along the osteogenic lineage. Interestingly, the results in vivo clearly showed that scaffolds with a triangular pore shape (which equals a layer-to-layer orientation of 60° during the plotting process) allowed significantly more bone formation compared to scaffolds with a rhombohedral pore shape (which equals 30°) [6].

The scaffolds manufactured for the in vivo study lacked to be volumetric (at least 10 × 10 × 10 mm^3^) [7], since the artificial defect size in small animal models only requires scaffolds of small dimensions and few layers. Those structures can be colonized by cells when they are seeded on top. In contrast, real implants have a higher volume and conventional cell seeding might only allow for cell colonization of the outer surface of the implant but not of its center, which is crucial for the successful regeneration of the defect in vivo. A possible solution for this problem is bioprinting, which enables the three-dimensional distribution of cells within scaffolds during the fabrication process. For this, cell carrier materials are needed; in combination with the embedded cells they form the bioink [8]. The CPC can be simultaneously processed with bioinks by multichannel plotting [9]. In order to tackle the problem of homogenous cell seeding of volumetric CPC implants, we investigated in this study whether a cell-laden fibrin hydrogel can infiltrate the pores between plotted CPC strands in order to distribute cells homogeneously within a scaffold structure. This approach can be refined toward a fully automated bioprinting process.

Non-autologous fibrin, acting as the second (cell carrier) material for the tissue engineered implants, was researched intensively as treatment material and as delivery system for cells and growth factors for the treatment of bone defects [10,11]. Fibrin, as well as its precursor fibrinogen, belong to the family of glycoproteins which are produced by mammals. They are crucial for wound healing by induction of blood clotting [12]. After an injury, several enzymes assemble at the wound site and activate thrombin which cleaves fibrinogen molecules that results in the formation of insoluble fibrin fibrils. Afterwards, the fibrils are covalently crosslinked by the fibrin stabilizing factor, a process which is mediated by calcium and thrombin [13]. Since fibrin holds a RGD-motif, cells being encapsulated in the fibrin polymer network can interact with it and attach to the RGD-domain. Fibrin glue is a standard material in surgeries for wound closure.

We hypothesized that the tissue engineered implants consisting of the plotted macroporous CPC structure filled with the cell-laden fibrin hydrogel accelerate the formation of bone tissue in vivo. Due to fast degradation of the fibrin, the embedded cells migrate towards the adjacent CPC strands and attach to their surface, thus, are placed efficiently and homogenously into the alveolar cleft where they promote new bone formation and defect healing. In this work, the CPC-fibrin biphasic implants were developed, the degradation behavior of fibrin in the presence of cells and CPC investigated and the cellular behavior within the two-material system in vitro characterized. Lastly, we report on the results of an in vivo pilot study in Lewis rats.

## 2. Results

### 2.1. Development of the CPC-Fibrin Implants

CPC scaffolds are osteoconductive but do not actively promote the growing of bone tissue. The integration of patient-derived cells, in line with the concept of tissue engineering, should increase the biological functionality of the constructs. Classical cell seeding may not allow for an efficient and homogeneous cell distribution within a plotted, volumetric construct of clinically relevant dimensions. Thus the simultaneous fabrication of CPC and cell-laden bioinks into one structure is a worthy approach enabling just that.

Both materials, CPC and fibrin, were purchased as clinically approved materials (see Section 4) and were not further modified. In pre-tests, we co-extruded the fibrinogen solution next to the CPC structure, followed by pipetting of thrombin solution onto the plotted structure to ensure formation of fibrin. In this approach, the fibrin gel successfully filled up the pores of a cubic-shaped CPC scaffold which had a strand-to-strand distance of 2 mm and outer dimensions of 12 × 12 mm^2^.

For the animal study presented in this manuscript constructs of a smaller scale were needed, which were not achieved successfully using a multichannel plotting process. Due to this reason it was decided to simplify the fabrication process for the further investigations while maintaining the material conditions as it would be in the real fabrication process. Therefore, sterilized CPC was plotted with a multichannel plotter in a laminar flow bench as a cylindrical scaffold (diameter 3.3 mm, height 0.5 mm, strand-to-strand distance 0.5 mm) with a layer-to-layer orientation of 60°, deduced from our previous study [6]. Subsequently, 15 µL fibrin precursor solution, consisting of 45.5 µg∙µL^−1^ fibrinogen and 25 U thrombin were pipetted onto the CPC scaffold. The solution filled up the pores of the CPC structure and covered it completely.

**Figure 1:**
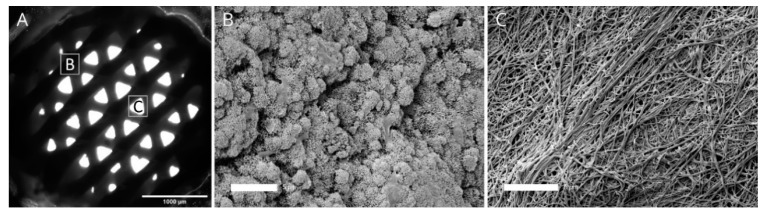
Brightfield image (**A**) of the biphasic calcium phosphate cement (CPC) -fibrin scaffolds with the positions of the subfigures B and C and the microstructure of CPC (**B**) and fibrin (**C**), observed by scanning electron microscopy. Scale bars: 1000 µm (**A**) and 5 µm (**B**,**C**).

After pipetting the fibrinogen and thrombin solution, scaffolds were incubated at 37 °C for 15 min to ensure formation of the fibrin network followed by immersion in cell culture medium. In presence of the forming fibrin gel, the CPC started to set transforming into nanocrystalline HAp of the CPC matrix. In the cell culture medium, the setting process proceeded. Figure 1A shows a microscopical brightfield image of the biphasic scaffold, where the fibrin hydrogel is not visible due to its translucent character but the pore structure of the plotted CPC layers can be observed. Further, we investigated the microstructure of CPC (Figure 1B) and fibrin (Figure 1C) by scanning electron microscopy. While CPC revealed a nanocrystalline structure, fibrin appeared as a fiber-like web.

### 2.2. Degradation Behavior of the Fibrin Hydrogel of the Cell-Laden Fibrin Hydrogel

#### 2.2.1. Degradation of Fibrin Dependent on Cell Species and Cell Number

**Figure 2:**
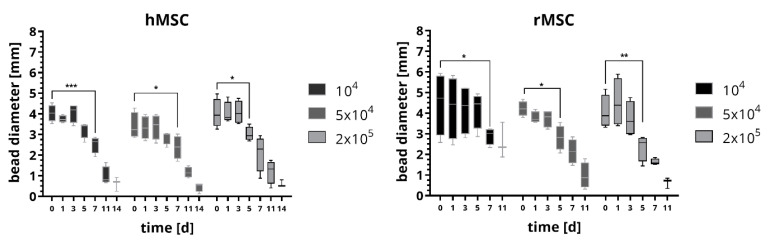
Degradation behavior of fibrin beads in presence of 10^4^, 5 × 10^4^ and 2 × 10^5^ human mesenchymal stem cell (hMSC) and rat mesenchymal stromal cells (rMSC), respectively. The diameter of the beads were determined from measurements in microscopic images. Statistical analysis using two-way ANOVA: incubation time has a significant influence with *p* < 0.001, the cell number has no significant influence for both, hMSC and rMSC; Tukey’s multiple comparison test: significant differences are plotted after the fibrin bead was significantly lower compared to day 0 for the first time. (*n* = 4, median ± min/max, * *p* < 0.05, ** *p* <0.01, *** *p* < 0.001).

Firstly, we examined the degradation properties of the fibrin network in absence of CPC but varied the cell density. Therefore we embedded 10^4^, 5 × 10^4^ and 2 × 10^5^ cells in fibrin beads with a volume of 15 µL and measured the average bead diameter in stereo-light microscopical images after incubation in cell culture medium over a period of 17 days (medium was exchanged every 3–4 days). Further we investigated the impact of the species using cells either from human or from rat. For human cells, an immortalized human mesenchymal stem cell (hMSC) cell line was used [14]; for rat cells, we isolated mesenchymal stromal cells (rMSC) from bone marrow harvested from the rat femur. The results are shown in Figure 2. For both, hMSC and rMSC, the average bead diameter decreased over time. The beads containing hMSC were stable for five days in case of 10^4^ and 5 × 10^4^ cells and three days for 2 × 10^5^ cells. Afterwards the beads started to degrade as indicated by a significant decrease in bead diameter; after 14 days, some beads were already dissolved and most beads had a diameter of less than 20% of the initial diameter. After an observation period of 17 days, no longer hMSC-laden beads were found in the cell culture medium. The cell number did not influence the degradation behavior significantly. Beads with embedded rMSC did not show distinct changes of the bead diameter for the first five days of culture for 10^4^ cells and three days for 5 × 10^4^ and 2 × 10^5^ cells, afterwards a strong degradation occurred and the bead diameter decreased significantly. Already after 11 days, some beads were dissolved and most beads showed diameters which were less than 20% of the initial diameter. After 14 days of cell culture, no longer beads containing rMSC were observed. Again, the number of rMSC did not significantly influence the degradation behavior of the fibrin beads. Remarkably, the fibrin beads with rMSC degraded faster compared to the fibrin beads with hMSC.

#### 2.2.2. Degradation of rMSC-Laden vs. Cell-Free Fibrin in Presence of a CPC Construct

**Figure 3:**
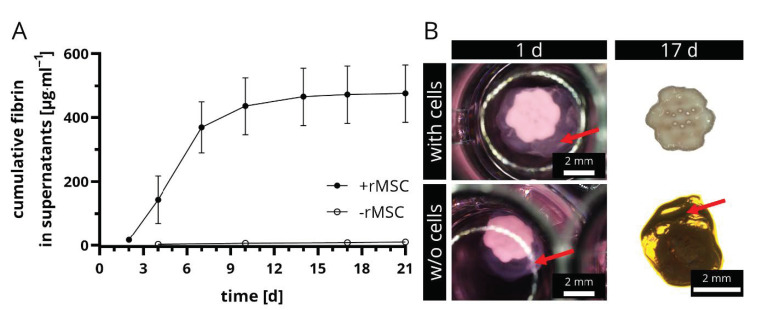
Degradation of fibrin in presence of CPC scaffolds. The concentration of fibrin degradation products in supernatants of rMSC-laden and rMSC-free biphasic CPC-fibrin constructs was measured over time, a cumulative release curve is shown (**A**) (mean ± SD, *n* = 5). Stereomicroscopical images clearly revealed that fibrin was only degraded in presence of rMSC, whereas cell-free scaffolds showed no signs of degradation (**B**). Red arrows tag the fibrin gel. Scale bars represent 2 mm.

In a second step, the degradation of rMSC-laden fibrin in presence of the CPC scaffold structure was investigated. Therefore, 2 × 10^5^ cells were encapsulated into the fibrin gel that was prepared on top of the CPC scaffold, thus the fibrin filled up the pores of the CPC scaffold. Cell-free biphasic scaffolds served as negative controls. The experimental setup allowed drawing conclusions about the role of cells in the constructs and the effect of the CPC structure on the fibrin degradation. Therefore, supernatants of these constructs were collected and the fibrin monomer content was determined via ELISA. The results as well as representative stereomicroscopical images of the biphasic structures with and w/o rMSC are shown in Figure 3A,B. Firstly, it is remarkable that the period of investigation was longer than in the previous experiment (21 days with CPC in comparison to only 14 days w/o CPC, see Figure 2). This implies that CPC has a stabilizing effect on rMSC-laden fibrin. The presence of cells was crucial for an ongoing degradation of the fibrin. Over the entire period of investigation, fibrin degradation products were measured in the supernatants in rMSC-laden samples, however more than 90% of the total measured fibrinogen amount was observed already after 10 days. In contrast, the cell-free negative controls did not show any relevant concentration in the supernatants (Figure 3A) and after 17 days, the fibrin on top of the CPC structure was still intact and comparable in size with those at the start of the experiment (Figure 3B).

### 2.3. Cell Survival and Migration in Biphasic CPC-Fibrin Constructs

**Figure 4:**
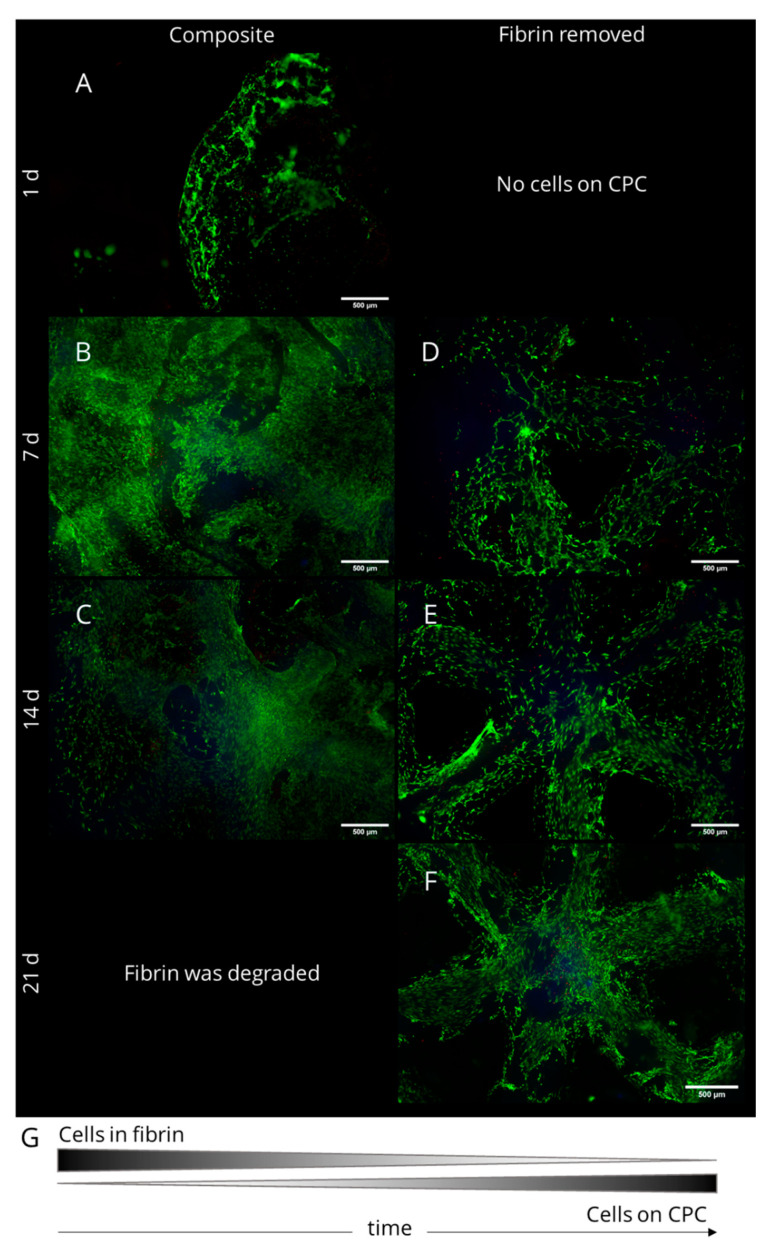
Fluorescence images of rMSC in biphasic CPC-fibrin scaffolds. Living cells are stained green, dead cells are stained red and CPC reveals a slight blue autofluorescence. With passing culture and fibrin degradation, the rMSC migrated onto the CPC surface. (**A**) after 1 day of cell cultivation on the composite scaffold; (**B**) after 7 days on the composite scaffold; (**C**) after 14 days on the composite scaffold; (**D**) after 7 days of cultivation and after removal of the fibrin; (**E**) after 14 days of cultivation and after removal of the fibrin; (**F**) after 21 days of cultivation and after removal of the fibrin. Scale bars represent 500 µm.

The response of rMSC in CPC-fibrin constructs was studied by encapsulating 2 × 10^5^ rMSC in 15 µL of fibrin which was pipetted onto freshly plotted CPC scaffolds. After 1,7, 14 and 21 days of cell culture, simultaneous staining of live and dead cells was applied to the composite constructs. Furthermore, the fibrin was removed gently from the structure to evaluate whether cells grow onto the CPC structure. Resulting fluorescence images are presented in Figure 4. Living cells appear green, the cell nucleus of dead cells is stained red and the CPC structure demonstrated a slight blue autofluorescence. After 1 day of cell culture, roundish rMSC were observed within the fibrin gel, some spread cells could be observed at the gel’s surface (Figure 4A). At the CPC surface, no rMSC were found. After 7 d, a dense cell network of spread cells was found at the surface of the fibrin and within the fibrin network. Furthermore, after gently removing the fibrin gel, cells with spread morphology appeared at the CPC surface (Figure 4B,D). A few dead cells were found in both, the fibrin gel as well as on top of the CPC surface. After 14 d, when the fibrin was degraded distinctly, many spread rMSC could be observed in fibrin regions close to the CPC, especially within the pores between the CPC strands (Figure 4C). In addition, distinctly more cells were found at the CPC structure compared to the previous time point (Figure 4E). The number of dead cells was negligible. After 21 d of culture, almost all fibrin was degraded. Compared to the previous time points, many rMSC were found on the CPC surface forming a dense cell network (Figure 4F). In conclusion, our investigation demonstrates that rMSC in biphasic CPC-fibrin scaffolds firstly spread within the fibrin matrix. With time and fibrin degradation, they migrate towards the CPC surface, where they attach and form a dense cell network. In the biphasic constructs, the fibrin hydrogel has been proven as a suitable cell delivery system for the CPC structure and eventually also for the surrounding defect area in vivo (Figure 4G).

### 2.4. In Vivo Pilot Study

#### 2.4.1. Study Design and Post-Operative Evaluation

Scaffolds were fabricated in sterile conditions in the dimensions like previously described [6]. Immediately after plotting CPC scaffolds with a layer orientation of 60°, 15 µL of rMSC-containing fibrin was pipetted on top (cell number: 2 × 10^5^). The cell-laden constructs were cultured in vitro for 7 days and then implanted into a bone defect in the palate of adult Lewis rats, following a surgical protocol developed in multiple studies before [6,15,16]. For this reason, we decided not to include a negative control allowing reduction of the animal number; in the previous studies, the negative control groups repeatedly provided similar results. Scaffolds were implanted with respect to the best fitting into the defect zone irrespective of caudal or cranial orientation. The healing times were either 6 or 12 weeks. The pilot study was completed by 12 out of 16 rats (survival rate of 75%). Four rats died during the healing time due to unknown reasons. In total, both time points, 6 and 12 weeks, could be completed by 6 rats each. The body weight of the adult rats was slightly increasing over time and no further complications were observed.

#### 2.4.2. Microcomputed Tomography and Histomorphology

**Figure 5:**
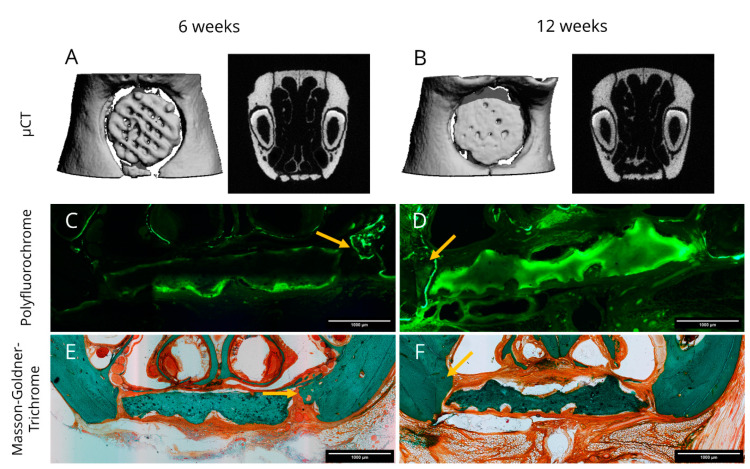
Representative images obtained from microcomputed tomography data (**A**,**B**) as well as fluorescence (**C**,**D**) and light (**E**,**F**) microscopy of histological sections of explants. (**A**,**C**,**E**) represent a healing time of 6 weeks and (**B**,**D**,**F**) a healing time of 12 weeks. Histological sections were labelled with polyfluorochrome dye (Calcein, **C**,**D**) prior to sacrifice and after microscopical analysis, Masson-Goldner-Trichrome stainings (**E**,**F**) were applied to the same sections. Arrows mark zones of newly formed bone tissue. Scale bars represent 1000 µm.

After sacrifice, all rats were investigated by µCT. Representative images of explants are presented in Figure 5A,B for 6 and 12 weeks, respectively. Lamellar and cancellous bone were isodense with the 3D plotted CPC structure. All reconstructions showed that the scaffolds stayed intact over the period of investigation; only in one case, a break was detected after 6 weeks. Two cranial located scaffolds dislocated slightly from the defect region.

New bone formation was assessed by polyfluorochrome labelling in vivo. Therefore, Alizarin and Calcein were injected seven and three days before sacrifice. Representative images for six and twelve weeks healing time are shown in Figure 5C,D. At both time points, only the Calcein label were detectable ex vivo. Osteogenesis could be confirmed at the borders of the defect location as well as in distant regions where the periosteum was not removed. That occurred to a greater extent when the scaffold had direct contact to the native bone. The scaffolds showed a pale green autofluorescence with label enhancement on their surface, which may be an indicator for local mineralization processes. Remarkably, the intensity of the calcein staining was generally more pronounced after twelve weeks compared to six weeks.

Furthermore, three out of six explants after twelve weeks revealed distinct morphological changes of the CPC scaffold structure resulting in strong convex and concave deviations that are clearly visible in Figure 5D,F indicating scaffold resorption.

Masson-Goldner-Trichrome stainings were applied to the histological sections afterwards. After 6 weeks a homogenous ossification starting from the defect margins into direction of the bone grafts was visible. The cone like structure of the newly formed bone was characterized by woven bone on the tip and lamellar bone on the basis adjacent to the former defect margin. The scaffold itself showed no morphological changes and the superficial pores were filled by non-mineralized tissue. As expected, fibrin was not detectable anymore after 6 weeks. (Figure 5E). At the end of the study, some scaffolds exposed a rough surface which might be a sign of resorption and they were deformed compared to the initial scaffold design (Figure 5F). Bone formation was ongoing, whereas no osseous integration of the scaffolds could be observed after 12 weeks. Fibrous tissue was clearly visible in the histological sections. The fibrous tissue located in-between the scaffold and the native bone revealed blood vessels. Further we observed that the respiratory nasal mucosa regenerated completely along the scaffold’s surface.

#### 2.4.3. Histomorphometry

**Figure 6:**
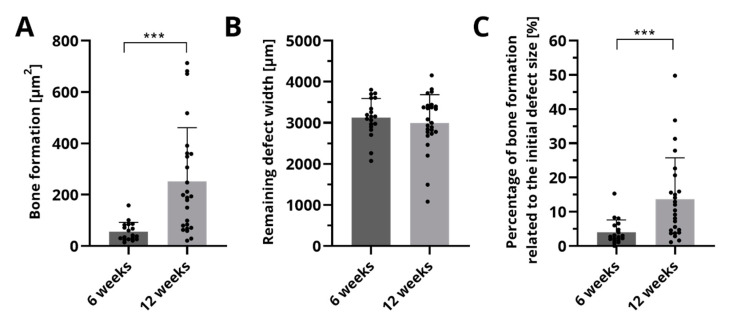
Histomorphometric analysis of bone formation (**A**), the remaining defect width (**B**) and the percentage of newly formed bone (**C**). Displayed are mean ± SD, *** *p* < 0.001, *n* = 19 and *n* = 25 for 6 and 12 weeks, respectively. The points mark the actual measured values of the histological sections.

The histological sections were analyzed quantitatively with respect to bone formation (Figure 6A), the remaining defect width (Figure 6B) and the percentage of bone formation related to the initial defect size (Figure 6C). After 6 weeks, an area of 52.6 ± 38.2 µm^2^ of newly formed bone was measured. Ongoing bone formation could be evidenced by a significant higher area of 251.6 ± 209.1 µm^2^ after 12 weeks. The remaining defect width did not change significantly over time. Related to the defect size, significantly more bone formation was observed after 12 weeks (13.7 ± 12.1%) compared to 6 weeks (4.0 ± 3.6%).

**Figure 7:**
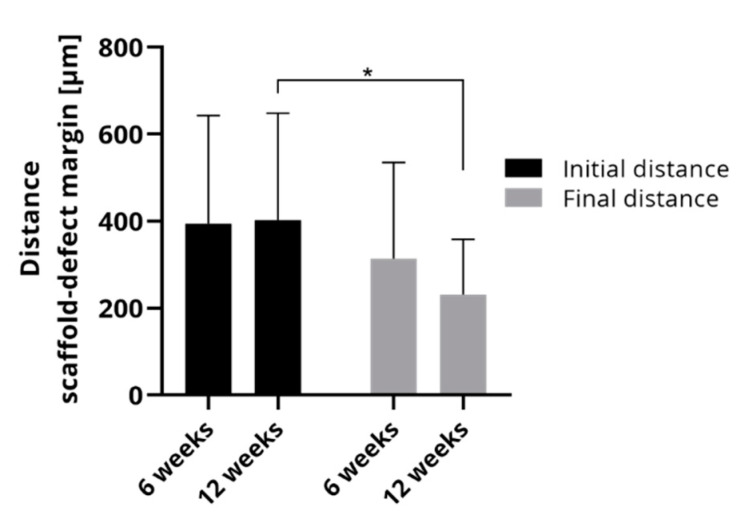
Average of initial and final distance of scaffold and defect margin of rats which were investigated either after 6 weeks or after 12 weeks (mean ± SD, * *p* < 0.05, *n* = 20 and *n* = 25 for 6 and 12 weeks, respectively).

Since bone formation occurs mainly from the defect margin in the direction of the scaffold, the comparison of initial and final distance of the scaffold and the defect margin allows us to draw conclusions about bone accrual. Figure 7 compares the initial and final distances in the histological sections of 6 weeks and 12 weeks, respectively. The initial distances were nearly identical between both groups implying that the artificial defects in the rats could be placed repeatedly without major deviations. The final distances between scaffold and defect margin were smaller than the initial distances. After 6 weeks, the final distance was 313.9 ± 221.1 µm and after 12 weeks, it could be determined as 231.12 ± 127.2 µm that was significantly less than the corresponding initial distance.

## 3. Discussion

Herein, we investigated the feasibility to combine a plottable CPC scaffold with an injectable cell-laden fibrin gel. Both components, CPC and fibrin, are clinically certified materials, which brings the novel biphasic constructs close to clinical application. In general, CPC can be plotted in any shape and dimensions using fugitive support gels that are plotted at the same time and washed away during post-processing [5]. With regards to the intended application of this manuscript it should be recognized that also constructs bridging real alveolar clefts in humans can be achieved by this process [6]. The fibrin used in this study was constituted from fibrinogen that was cleaved by thrombin. Whereas a fibrin gel would not have been plottable, its fibrinogen precursor was already shown to be processable with bioprinting technologies [17,18]. In our approach, the fibrinogen solution was supposed to fill up the pores between the CPC strands, similarly as described for other soft/hard biomaterial combinations before [19]. Han et al. printed stiff poly-ε-caprolactone in combination with a fibrinogen-based bioink, evidencing the suitability of this concept [20]. In our study, the fibrinogen solution was pipetted onto the unset CPC structure. After plotting of the CPC scaffolds, they were shielded from humidity, otherwise the setting process would have been initiated. In this case, the interaction of CPC and the cell-laden fibrin would have been different from those in the fully bioprinted construct and from those observed in our investigations. Due to this, the results of this study are valid for fully biofabricated CPC-fibrin constructs. Volumetric, biphasic constructs of clinically relevant size likely can be fabricated as shown previously [21]. Finally, a novel type of scaffold consisting of cell-laden fibrin and CPC was fabricated precisely fitting into an artificial alveolar defect.

CPC-fibrin composites have been previously investigated in several studies. Cui et al. used fibrin glue as a liquid phase that was mixed with the precursor powders obtaining an injectable CPC with a setting time of approx. 10–20 min. The fibrin in the composite elevated the osteogenic differentiation of human bone marrow stem cells (hBMSCs) compared to a water-based CPC [22]. Next to the high osteogenic potential of these grafts, they were later shown to promote blood vessel formation in vivo due to the presence of fibrin [23]. Another approach was investigated by Song et al., who fabricated a composite of a powder-liquid CPC with alginate-fibrin hydrogel microfibers. HBMSCs that were encapsulated into the microfibers were released after fast degradation of the hydrogel phase and promoted significantly more bone formation compared to a cell-free control group [24]. Recently, our group bioplotted a plasma-derived bioink containing fibrin next to CPC strands. This allowed for the spatial distribution of osteoprogenitor cells that could proliferate tremendously and differentiate along the osteogenic lineage in the bioink compared to a fibrin-free control [21]. All these studies evidence that the presence of a cell-laden fibrin hydrogel in CPC-based constructs evokes beneficial effects for both, cell seeding and osteoblastic differentiation in vitro and in vivo.

A crucial aspect of tissue engineered constructs is their degradation behavior in vivo. The HAp-forming CPC constructs can be resorbed by osteoclasts which is a slow process [25,26]. Fibrin gels are insoluble and thus cannot be dissolved. However, it is cleaved in presence of fibrinolytic enzymes, such as urokinase, plasmin or nattokinase; a process that is crucial for the wound healing process [27,28].

The results of the degradation studies in this work can be summarized as follows: (i) Both tested cell types, hMSC and rMSC, are able to secrete fibrinolytic enzymes which result in the degradation of fibrin. (ii) The number of encapsulated cells had no effect on the degradation. (iii) Fibrin beads with rMSC were degraded faster compared to those with hMSC. (iv) CPC constructs act slightly stabilizing for rMSC-laden fibrin gels.

Our experiments clearly prove that rMSCs are able to degrade human fibrin, a finding that was reported similarly by Mehanna et al. [29] It is surprising that rMSC evoked a faster degradation of the fibrin beads compared to hMSC. Even though the species-related differences may explain this effect, it should also be considered that the rMSC are a heterogenous population of bone marrow cells whereas the hMSC were single cell-derived and proven for its stem cell character [14] and thus cellular interactions and activities are different. However, to the best knowledge of the authors, such a comparison was not investigated before and it needs further studies to evidence this effect. Remarkably, the cell number had no significant effect for both cell types. Even the lowest cell number (10^4^ hMSC and rMSC) was sufficient to degrade the applied amount of fibrin. One would expect that the highest cell number leads to the fastest degradation. It remains speculative but it seems that cells, that are able to spread within the gel and thus form a dense cell network (enabling communication between the cells), self-regulate the expression of fibrinolytic enzymes. This means, that cells in a low density might need to be more active to communicate with each other leading to a higher cellular activity and thus upregulated expression whereas cells in a high density are less active and thus show a downregulated expression of the enzymes. Shamsul et al., observed that fibrin gels with a low density of chondrocytes showed a stronger decrease of the bead diameter compared to fibrin gels with a high chondrocyte density [30]. Although the effect was overlayed by expression of cartilaginous extracellular matrix in the high cell density group, this observation confirms our finding, that the cell number plays only a minor role for fibrin degradation. The fibrin gel was longer stable in presence of the plotted CPC structure. The positive role of calcium during fibrin formation [31] and its impact on the downregulated activity of fibrinolytic enzymes [32] have been studied for a long time. Although the calcium-deficient HAp matrix of the set CPC structure is reportedly absorbing calcium ions from its environment [33], it is probable that the calcium ions at the surface of the construct are interacting locally with the fibrin gel in a stabilizing manner. This phenomenon was also observed when alginate-based inks were biofabricated in combination with CPC and could be stacked into multiple layers due to local ionic crosslinks of the alginate at the CPC-bioink interface [9,21]. The only local interaction also might explain why the positive effect of CPC on the fibrin stability was not more pronounced. In constructs of greater dimensions, the same interactions between cells and materials are expected. However, it must be considered that bioprinted cells in the center of a volumetric, bioprinted scaffold are more distant to the host bone than in our defect model, which could influence their supply with nutrients and oxygen, which could influence the bone healing. Summarized, our observations in vitro demonstrate that fibrin finally gets degraded in biphasic CPC-fibrin constructs and that embedded cells are migrating onto the CPC structure evidencing the suitability of fibrin to act as cell delivery system.

The quantitative results of the in vivo pilot study, namely the bone formation, the percentage of newly formed bone in the defect area and the final distance of the scaffold to the defect margin, showed ongoing bone formation and osseous healing over 12 weeks. Particularly in the second period of investigation (weeks 6 to 12 after implantation), a significant increase of bone healing was observed. In our previous study [6], the bone formation of 3D plotted CPC scaffolds was evaluated in the same animal defect model. The outer and inner structural design of the CPC scaffolds was the same. Due to these reasons, the results obtained in this work can be compared to the results from the previous study in which rMSCs were seeded in conventional manner directly onto the CPC scaffolds. The resulting tissue engineered constructs led to a bone formation of 7.7% after 6 weeks and 8.7% after 12 weeks [6]. This means, that the direct seeding of cells led to the formation of bone tissue in the first 6 weeks but in the weeks 6 to 12 no significant further bone formation was observed. This is clearly contrary to the results obtained using a fast degrading fibrin hydrogel as a cell delivery system. This approach led to bone formation especially in the second healing period and further the final bone formation was distinctly higher using fibrin (13.7% after 12 weeks). Xu et al., discussed recently that cell delivery vehicles such as cell-laden hydrogels are advantageous compared to direct seeding of CPC structures, which is in line with our observations [34].

However, in the previous study [6] also an empty control defect was analyzed, leading to a bone formation of 13.2% after 6 weeks and 22.5% after 12 weeks. The remaining defect width measured 2.74 ± 0.14 mm (6 weeks healing time) and 2.26 ± 0.14 mm (12 weeks). From a clinical point of view, it has to be noticed, that empty defects representing not the clinical reality, because alveolar clefts were always augmented and mostly autografts were applied. Unfortunately, in small animal model like rats a defect augmentation with autologous bone grafts harvested from iliac crest are not realizable. This comparison would be aim of further studies in large animal models. The comparison of tissue engineered bone grafts with a cell-free group (plotted CPC with 60° strand rotation only) was included in our previous study [6]. Pure CPC scaffolds led to the highest bone formation of the scaffold groups so far (8.2% after 6 weeks and 19.0% after 12 weeks), with ongoing bone formation in both healing periods. The remaining defect width of pure CPC scaffold was 2.78 ± 0.14 mm after 6 weeks and 2.29 ± 0.14 mm after 12 weeks. This observation questions the potency of tissue engineered applications for the treatment of alveolar clefts—at least in the rat model: So far, the role of cell seeding of bone grafts applied to the defect model was not conclusive [6,15,16]. A main feature of this model is the small height of the defect margin, which measures less than 0.5 mm and this may handicap the initial stable fixation of the bone graft and additionally a mechanical load cannot be excluded due to the nutrition behavior of the rats. In a later clinical application, the geometry of a human alveolar cleft would lead to a higher initial scaffold-to-bone contact area and a reduced potential for mobility of the bone graft. Another reason for this could be that the defect zone in the rat model is a rather static system without blood exchange or any dynamic exchange of body solutions. This impairs the precipitation of HAp crystals on the CPC surface, which is known to be bioactive and further oxygen and nutrient supply to cells is limited. In this light, the positive results obtained in this study even can be considered as promising. Due to this reason, in a next step the investigation of the biphasic CPC-fibrin constructs in another alveolar cleft defect model of a bigger animal is aspired, verifying the findings of this study and answer the remaining questions.

A crucial point for the application of engineered bone grafts for cleft palates is their acceptance by the patient. A plain CPC structure is not expected to promote fast healing of the native bone tissue, [26,35] therefore tissue engineering strategies should still be taken into account for these kind of defects. Since the volumetric implants cannot be easily functionalized by direct seeding with cells, using fibrin as cell delivery system could be a promising alternative, which can be realized by biofabrication technologies. This allows for the fabrication of defect shape-specific implants consisting of clinically certified materials (CPC and fibrin) with cells directly derived from the patient. These highly specified constructs could be a relevant new form of regenerative therapy for the treatment of cleft lips and cleft alveoli.

## 4. Materials and Methods

### 4.1. Fabrication of CPC-Fibrin Implants

CPC was obtained from INNOTERE (Radebeul, Germany) and sterilized by γ-irradiation at 25 kGy. Fibrin (Tisseel, Baxter, Westlake Village, CA, USA) was bought as two component material which was mixed from human fibrinogen and human thrombin. Both materials were used as clinically certified, ready-to-use materials without further modification.

First, CPC scaffolds were fabricated in a laminar flow bench using a multichannel plotter (BioScaffolder 3.1, GeSiM, Radeberg, Germany). The CPC was plotted with a velocity of 12 mms^−1^ with an applied air pressure of 150 kPa using a conical steel needle with an inner diameter of 230 µm (Globaco, Rödermark, Germany). The scaffold’s geometry was as follows: cylindrically shaped scaffolds had a diameter of 5 mm (in vitro cell studies) or 3.3 mm (in vitro degradation study and in vivo study), a height 0.48 mm with a layer height of 0.12 mm and a layer-to-layer orientation of 60°. The strand distance was set as 0.5 mm (degradation study and in vivo study) and 2 mm (in vitro cell study).

In a second step, 7.5 µL fibrinogen and 7.5 µL thrombin solution were pipetted onto the unset CPC scaffolds forming a fibrin gel. The construct were allowed to rest at 37 °C for 15 min. Afterwards, CPC-fibrin constructs were incubated in aqueous cell culture medium.

### 4.2. Cell Culture

#### 4.2.1. Cell Isolation and Expansion

Two different types of cells were used: A human mesenchymal stem cell line, immortalized with telomerase reverse transcriptase (hMSC) [14] and primary rat mesenchymal stromal cells (rMSC). HMSC were expanded in cell culture medium (α-MEM with 10% fetal calf serum and% penicillin/streptomycin), the cell culture medium was exchanged twice a week. rMSC were isolated from the bone marrow of Lewis rats as described previously [16]. In brief, bone marrow aspirate of the rat’s femur was centrifuged for 10 min at 1200 rpm; the resulting pellet was transferred to cell culture flasks. Then rMSC were expanded in cell culture medium until passage 3 for use in the experiments.

#### 4.2.2. Degradation Experiments

The degradation of cell-laden fibrin was investigated in presence and absence of freshly plotted, unset CPC structures. For the first experiment (Section 2.2.1), hMSC and rMSC were trypsinated, counted and subsequently mixed with 7.5 µL of fibrinogen precursor solution. The degradation was characterized in dependence of three different cell numbers in the fibrin beads (*n* = 4 for each cell number): 10^4^, 5 × 10^4^ and 2 × 10^5^. The cell-containing solution was pipetted into well plates which were covered with hydrophobic parafilm. Directly after pipetting, 7.5 µL of thrombin solution were mixed thoroughly with the fibrinogen achieving cell-laden fibrin beads. The degradation was studied by measuring the bead diameter in stereo microscopical images (using a Leica M205 C stereo microscope equipped with DFC295 camera, Leica, Germany) taken at several time points of incubation. Cell culture medium was exchanged ever 3-4 days over the entire period of investigation.

For the second experiment (Section 2.2.2), 2 × 10^5^ rMSC were encapsulated in fibrinogen solution, pipetted on top of freshly plotted CPC structures, subsequently followed by a thorough mixing with thrombin in order to achieve fibrin gels that were filling up the pores between the plotted CPC strands. The fibrin content was determined in cell culture supernatants of the samples (*n* = 5) at certain time points (2, 4, 7, 10, 14, 17 and 21 days; the cell culture medium was exchanged completely)) using a human fibrinogen ELISA kit (ab108841, abcam, Cambridge, United Kingdom) following the manufacturer’s protocol. Cell-free CPC-fibrin scaffolds (*n* = 3) served as negative controls.

#### 4.2.3. Cellular Behavior

The behavior of cells in the biphasic CPC-fibrin was investigated microscopically by evaluation of fluorescence images using a Keyence BZ-X800 fluorescence microscope (Keyence, Osaka, Japan). Therefore, rMSC-laden CPC-fibrin constructs were fabricated as described before. After 1, 7, 14 and 21 days of culture, living rMSC were stained with Calcein AM and dead rMSC were stained with Ethidium homodimer-1 (both part of the LIVE/DEAD Viability/Cytotoxicity Kit, for mammalian cells, ThermoFisher Scientific) following the manufacturer’s protocol. Firstly, the intact biphasic structure was imaged with both, fibrin and CPC. After the imaging, the fibrin layer was gently scraped with a spatula until the CPC surface was completely free. This allowed for investigations of the cell viability but also for localization of the rMSC and gave insights into the morphology of living cells.

### 4.3. In Vivo Application in a Rat Alveolar Cleft Model

The animal study was approved by the Commission for Animal Studies at the District Government Dresden, Germany (DD24-5131/354/26). 16 adult male Lewis rats (Janvier Labs, Le Genest-Saint-Isle, France) with an average body weight of 450 g and an age of 6 months at the beginning were used. All rats were housed according to the current regulations in a light- and temperature-controlled environment. They had access to water ad libitum and were fed with pellets (ssniffSpezialdiäten GmbH, Soest, Germany). The rats were randomly divided into the 2 experimental groups. The animals were anaesthetized by intraperitoneal injection of ketamine (100 mg/kg body weight) and xylazine (10 mg/kg body weight) and fixed in a dorsal position. An artificial alveolar cleft with a diameter of 3.3 mm was created surgically in the anterior maxilla of each animal as described previously [6]. Each rat received one bone graft. Postoperatively, the animals received amoxicillin trihydrate (Fort Dodge Veterinär GmbH, Würselen, Germany) 15 mg/kg body weight once and 4 mg/kg body weight carprofen subcutaneously (Rimadyl; Pfizer Deutschland GmbH) every 24 h for 4 days. The animals were fed with a soft diet for the first 3 days and, subsequently, received a regular diet. Postoperatively, the animals and their behavior were monitored and the body weight was measured every 2 weeks. For the ex vivo assessment of the dynamic bone formation, all rats received intraperitoneal injections of the fluorochrome dyes Alizarin (30 mg/kg body weight) and calcein (20 mg/kg body weight) 7 and 3 days prior to sacrifice.

### 4.4. Evaluation Methods

After sacrifice, the cranium of each rat was dissected and fixed in 4% formaldehyde. Microcomputed tomography and preparation of the histological samples followed.

#### 4.4.1. Microcomputed Tomography

3D-µCT was performed ex vivo with a cone beam-based µCT (VivaCT40, SCANCO Medical AG, Brüttisellen, Switzerland) at a *x*-ray energy of 70 kVp and a voltage of 114 mA. The voxel size was set as 30 µm. The integration time was 200 ms. After 3D reconstruction of the defect area, the fitting accuracy of the CPC-fibrin scaffolds was assessed descriptively.

#### 4.4.2. Histology and Histological Analysis

Samples were dehydrated in a graded series of ethanol and embedded in methylmethacrylate (Technovit^®^ 9100, HeraeusKulzer, Wehrheim, Germany) as described previously [15], followed by production of coronal sections which were produced according to Donath’s sawing and grinding technique [36]. This allowed for achieving 3–4 central sections of each specimen that were used for subsequent evaluation. After polishing, fluorochrome markers were analyzed followed by Masson-Goldner trichrome stainings. The obtained sections were imaged by fluorescence microscopy. After Masson-Goldner trichrome staining, light microscopy was performed. Both investigations were conducted using an Olympus BX 61 microscope (Olympus Deutschland GmbH, Hamburg, Germany) with cell^F Imaging Software for Life Science (Olympus). Multiple image alignment was performed using an automatic scanning table (Märzhäuser, Wetzlar, Germany). Thus, 8 images per sample were scanned with a 10 × 10-fold magnification and manually fused to one image.

Firstly, the dynamics of bone formation was assessed by analysis of fluorochrome marker uptake. This allowed drawing conclusions of the direction and distribution of bone formation. After imaging, Masson-Goldner trichrome stainings were applied to the sections. Then, the position of the scaffold, its surface, the interactions between host bone and bone graft as well as the bone formation on the defect margins were evaluated descriptively.

Quantitative measurements in the images were performed to obtain parameters characterizing osseous healing, knowing that the complexity of 3D bone formation cannot completely described by formulae. Therefore, the remaining defect width (Equation (1)), bone formation in the defect area and the percentage of the newly formed bone relating to the particular initial defect area (Equation (2)) were determined. Additionally, the lowest distance between initial or final defect margin and scaffold were measured on both sides of the bone graft. All measurements were realized by one examiner who was masked regarding to the experimental groups.

(1) $$\mathrm{remaining}\;\mathrm{defect}\;\mathrm{width}=\;\frac{{\mathrm{distance}}_{\mathrm{defect}\;\mathrm{margins}\;\mathrm{cranial}}+{\mathrm{distance}}_{\mathrm{defect}\;\mathrm{margins}\;\mathrm{caudal}}}{2}$$

(2) $$newly\;formed\;bone=\;\frac{new\;formed\;bone\;area}{initial\;osseous\;defect\;area}\times 100\%$$

### 4.5. Statistics

Data obtained in vitro were tested with a two-way ANOVA followed by Tukey’s multiple comparison test. The resulting measurements of bone formation, the remaining defect width and the percentage of newly formed bone were statistically tested for significance using the Welch’s t-test. The differences of initial and final distance of scaffold and defect margin were tested with a two-way ANOVA followed by Sidak’s multiple comparison test. Significant differences were assumed for *p* < 0.05. All tests were performed with GraphPad Prism version 8 software (GraphPad Software, La Jolla, CA, USA).

## 5. Conclusions

3D printing of a clinically certified calcium phosphate cement paste in combination with MSC-laden fibrin, that is also certified for clinical use is suitable for the fabrication of scaffolds which fit exactly into an artificial alveolar defect at the time point of surgery. In vitro studies showed that the MSC-laden hydrogel acted as cell delivery system where the cells migrated onto the CPC structure with ongoing degradation of the fibrin. A pilot study in vivo revealed that the bone formation increased significantly on the defect margins but that the constructs were not completely osseous integrated. However, in the light of the application in the patient, fibrin gel could be a relevant delivery system for cells into the defect region. The creation of a 3D plotted and tissue engineered bone graft for alveolar cleft osteoplasty could preserve patients from donor site morbidity.

## Data Availability

The datasets generated for this study are available on request to the corresponding author.

## Abbreviations

CPC	Calcium phosphate cement
HAp	Hydroxyapatite
hMSC	Human mesenchymal stem cells
rMSC	Rat mesenchymal stem cells
